# How can MRI descriptors be optimally combined to predict idiopathic intracranial hypertension?

**DOI:** 10.1093/bjr/tqaf080

**Published:** 2025-04-15

**Authors:** George G Bitar, Philip Touska, Ata Siddiqui, Joshua Harvey, Ndidi Edi-Osagie, Haziq Chowdhury, James McHugh, Eoin O’Sullivan, Steve Connor

**Affiliations:** Department of Radiology, University College London Hospitals NHS Foundation Trust, London, NW1 2BU, United Kingdom; Department of Radiology, Guy’s and St Thomas’ Hospitals NHS Foundation Trust, London, SE1 9RT, United Kingdom; Department of Radiology, Guy’s and St Thomas’ Hospitals NHS Foundation Trust, London, SE1 9RT, United Kingdom; Department of Radiology, King's College Hospital NHS Foundation Trust, London, SE5 9RS, United Kingdom; Department of Ophthalmology, Moorfields Eye Hospital NHS Foundation Trust, London, EC1V 2PD, United Kingdom; Department of Radiology, Manchester University NHS Foundation Trust, Manchester, M13 9WL, United Kingdom; School of Biomedical Engineering and Imaging Sciences, King's College London, London, SE1 7EH, United Kingdom; Department of Ophthalmology, University Hospitals Sussex NHS Foundation Trust, Sussex, BN2 5BE, United Kingdom; Department of Ophthalmology, King's College Hospital NHS Foundation Trust, London, SE5 9RS, United Kingdom; Department of Ophthalmology, King's College Hospital NHS Foundation Trust, London, SE5 9RS, United Kingdom; Department of Radiology, Guy’s and St Thomas’ Hospitals NHS Foundation Trust, London, SE1 9RT, United Kingdom; Department of Radiology, King's College Hospital NHS Foundation Trust, London, SE5 9RS, United Kingdom; School of Biomedical Engineering and Imaging Sciences, King's College London, London, SE1 7EH, United Kingdom

**Keywords:** case control study, neurology, central nervous system, magnetic resonance imaging, logistic regression, idiopathic intracranial hypertension

## Abstract

**Objectives:**

To establish how MRI descriptors on standard MRI sequences can be optimally combined to predict idiopathic intracranial hypertension (IIH).

**Methods:**

A retrospective single-institution cross-sectional study evaluated consecutive IIH patients undergoing MRI between 2002 and 2015 and a control group. Six established and 8 exploratory MRI descriptors were evaluated. Two observers independently analysed MRI descriptors on T1w sagittal and T2w axial sequences while blinded to clinical data with consensus obtained. Inter-rater reliability was calculated, and the presence of MRI descriptors was compared between IIH patients and controls (Bonferroni correction, *P* < 0.004). Forward stepwise logistic regression determined which combination of MRI descriptors best predicted IIH.

**Results:**

Fifty-four IIH patients (mean age 31.2, standard deviation 10.2, 3 men) and 54 control subjects (mean age 31.7, standard deviation 7.1, 3 men) were evaluated. There was excellent inter-rater reliability for 13/14 MRI descriptors. There were 4/6 established and 6/8 exploratory MRI descriptors associated with IIH (*P* < 0.004). The optimal combination of descriptors to predict IIH was vertical tortuosity of the optic nerve, enlarged optic nerve sheath, globe flattening score ≥ 2, Yuh score ≥ 3, cervical skin folding, and cervical fat thickness ≥ 10.5 mm. The model correctly classified 93.5% of cases (sensitivity 94.4%, specificity 92.6%, area under the receiver operating characteristic curve [AUC] 0.965).

**Conclusions:**

Evaluating a combination of vertical tortuosity of the optic nerve, enlarged optic nerve sheath, globe flattening, Yuh score, cervical skin folding, and cervical fat thickness optimally predicts IIH.

**Advances in knowledge:**

New MRI features are validated for the diagnosis of IIH and the optimal combination for diagnosis is established.

**Registration number:**

N006 (local institutional review). The full study protocol can be requested from the corresponding author.

## Introduction

Idiopathic intracranial hypertension (IIH) is characterized by an unexplained elevation of intracranial pressure (ICP) with normal cerebrospinal fluid (CSF) constituents and no evidence of either structural lesion, hydrocephalus, or meningeal abnormality on neuroimaging. Clinical presentations include headaches, tinnitus, and visual symptoms. It predominantly presents in young-middle-aged females with the prevalence (76/100 000) and incidence (7.8/100 000/year) having considerably increased over the past decade in line with increases in body mass index (BMI).^1^

Various MRI features have been shown to be associated with IIH including an empty sella, posterior globe flattening, optic nerve sheath distension or tortuosity, slit-like ventricles, inconspicuous subarachnoid spaces, and cerebellar tonsillar descent. These MRI descriptors were the subject of a systematic review and meta-analysis which calculated a pooled sensitivity of these descriptors ranging from 6.1% to 68.6% and with a specificity ranging from 84.0% to 99.2%.^2^ The most diagnostically useful imaging feature is known to be bilateral transverse sinus stenoses with a sensitivity of 84.4% and a specificity of 94.9%.^2–4^

Since these MRI descriptors may also be present in asymptomatic individuals and clinical features of IIH may be nonspecific or unreported, understanding the significance of these imaging features can be a challenge during routine reporting. Further work is required to determine how to best interpret these MRI descriptors, with a particular focus on those applied to standard intracranial MRI protocols. In order to better distinguish patients with IIH, it is also useful to investigate new established MRI descriptors and to optimize diagnostic performance by evaluating combinations of MRI descriptors.

The objectives were to evaluate a range of established and exploratory MRI descriptors on T2w axial and T1w sagittal brain sequences for their ability to distinguish IIH patients from controls. Furthermore, it was aimed to determine the optimal combination of MRI descriptors for the prediction of IIH.

## Methods

### Patients

This was a retrospective, case-control single-institution study with local institutional approval (project reference 006) and no consent required from patients.

A search of the neuro-ophthalmology database was performed to identify patients with IIH according to the modified Dandy criteria^5^ (Table 1), and this was cross referenced to the radiology information system (CRIS, Healthcare software solutions Ltd, Mansfield, UK) to determine consecutive patients undergoing MRI between 2002 and 2015. Patients were excluded if lumbar puncture data were not available within a year of the MRI, MRI sequences were degraded, or if therapeutic shunting was performed prior to MRI.

**Table 1.** Modified Dandy criteria.

Modified Dandy criteria
Signs and symptoms of increased intracranial pressure (headaches, nausea, vomiting, transient visual obscurations, papilloedema)
No localizing neurologic signs otherwise, with the single exception being unilateral or bilateral VI nerve paresis
CSF can show increased pressure, but no cytologic or chemical abnormalities otherwise
Normal to small symmetric ventricles must be demonstrated (originally required ventriculography, but now demonstrated by CT)

Abbreviation: CSF = cerebrospinal fluid.

**Figure 1.**
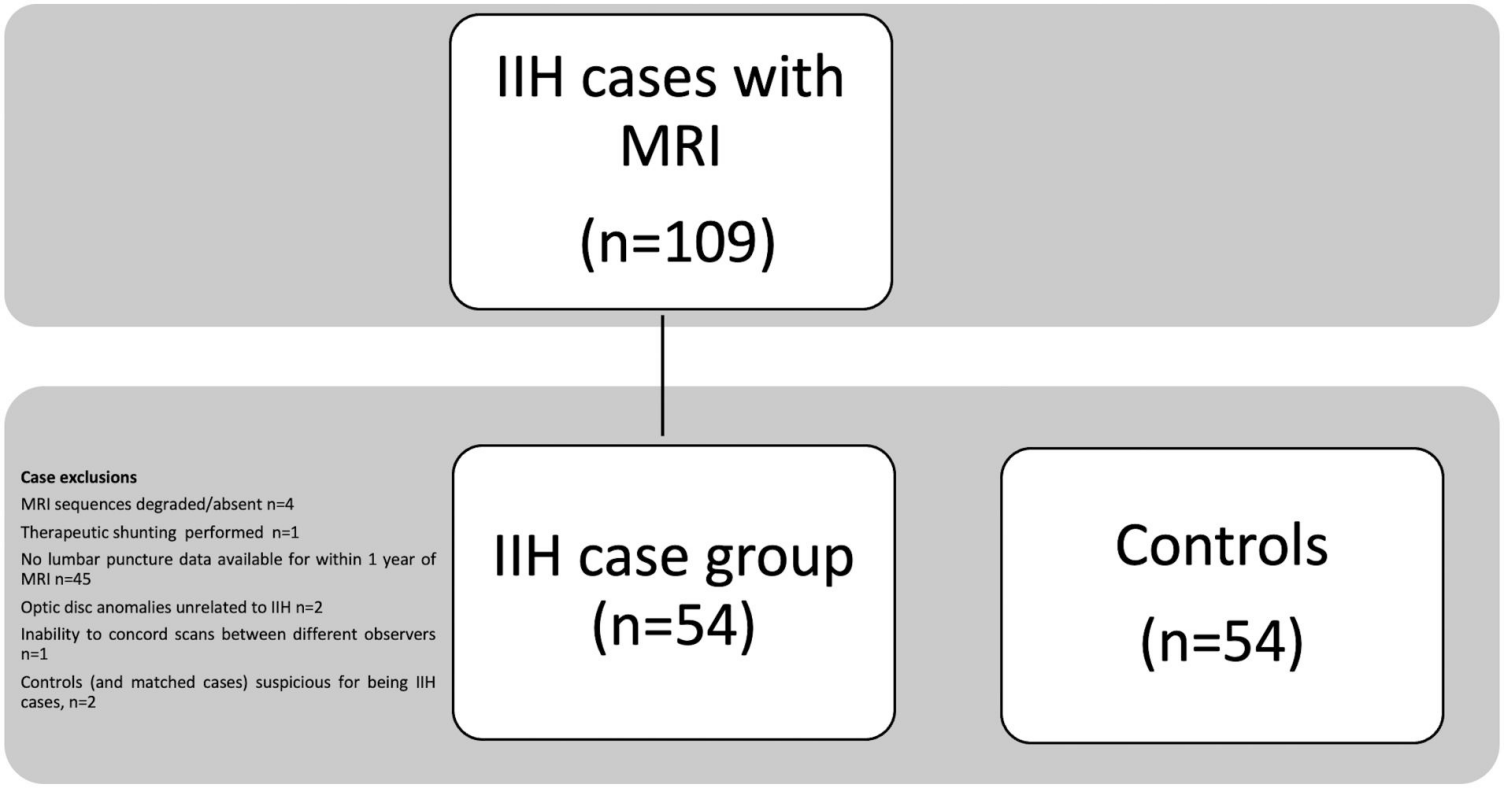
Flow chart demonstrating case exclusions and the final case and control groups.

An age- and sex-matched control group was selected using the radiology information system to identify consecutive patients undergoing MRI for seizure investigation and electronic medical records were reviewed for clinical parameters (Table 2). Exclusion criteria were any documented clinical or imaging features which could be associated with raised ICP or altered CSF dynamics including hydrocephalus, space-occupying lesion, venous sinus thrombosis, cerebral herniation, and Chiari malformations. Figure 1 demonstrates the patient selection.

**Table 2.** Demographics of the IIH and control groups.

Demographic	IIH	Control
Age (mean, SD, range)/years	31.2, 10.2, 15-54	31.7, 7.1, 17-41
Male:Female ratio	3:51	3:51
Weight (mean, SD)/kg	96.3, 18.8	Not available
Mean CSF opening pressure/cmH_2_0	38.2	N/A
Headache/%	81.5	N/A
Tinnitus/%	38.9	N/A
Transient visual obscurations/%	25.9	N/A
CSF leak/%	1.9	N/A
Treated with acetazolamide/%	85.2	N/A

Abbreviations: CSF = cerebrospinal fluid; IIH = idiopathic intracranial hypertension.

#### MR imaging

##### Protocol and analysis

Imaging was performed on Siemens Aero 1.5 T (Siemens, Erlangen, Germany) and General Electric Signa HDx 1.5 T (GE Healthcare, Milwaukee, Wisconsin) MRI systems. Equivalent MRI sequences were anonymized and randomly assigned to a single research worklist for both IIH patients and controls. Only T2w axial (4/0 mm) and T1w (4/0 mm) sagittal images were made available for review (Table 3). Two subspecialty radiologists (6 and 20 years’ experience respectively) evaluated MRI descriptors while blinded to clinical data, with consensus achieved for any discrepancies.

**Table 3.** Basic magnetic resonance imaging protocol for the assessment of MRI descriptors.

Sequence	Plane	Slice thickness/gap (mm)	TR/TE	Field of view (mm)	Number of averages	Pixel Bandwidth (Hz/pixel)	Flip angle (degrees)	Acquisition matrix
T1w	Sagittal	4/0	549/11	220 × 220	1	200	160	384/269
T2w	Axial	4/0	5830/102	220 × 220	1	190	150	384/346

##### Selection of MRI descriptors

**Figure 2.**
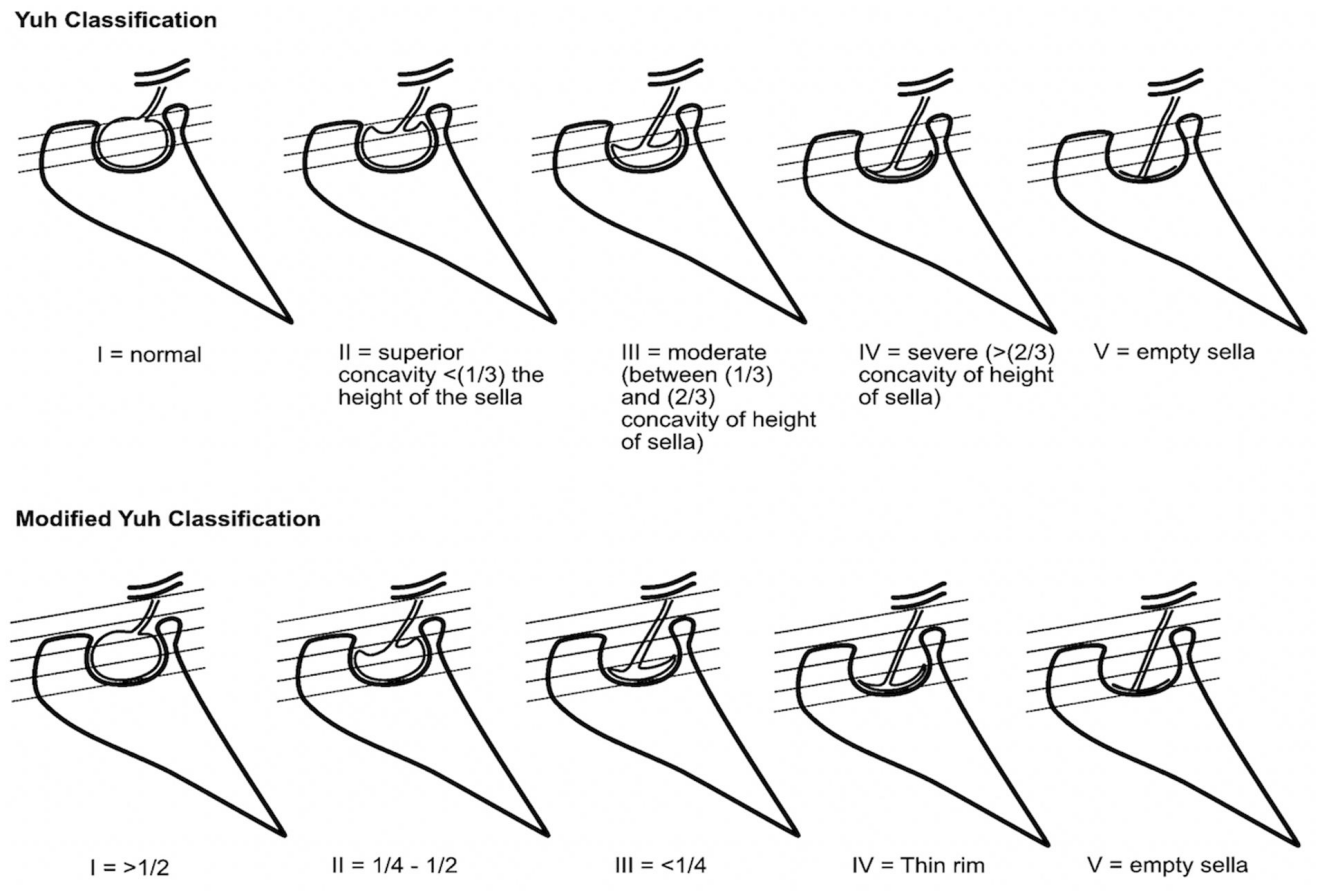
Classification of Yuh and modified Yuh scores. Abbreviation: IIH = idiopathic intracranial hypertension.

**Figure 3.**
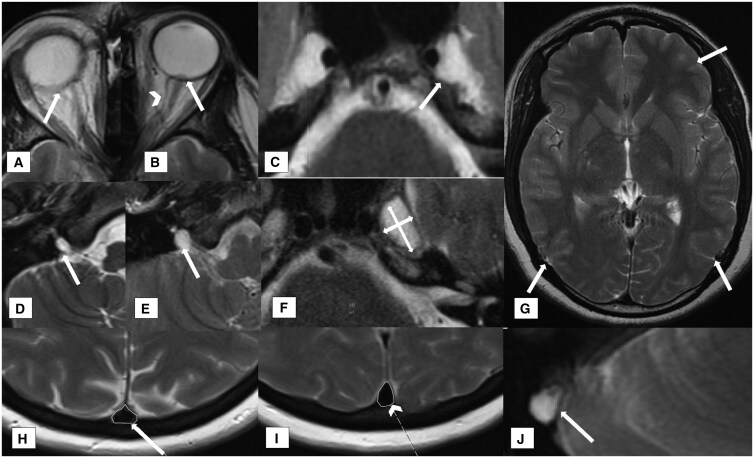
MRI features scored on T2w axial imaging and illustrated in a patient with IIH. (A) Grade 2 globe flattening (white arrow). (B) Grade 3 globe flattening (white arrow) and enlarged peri-optic CSF spaces (arrowhead). (C) Enlarged Meckel’s caves with focal meningocele extending beyond the margin of Meckel’s cave (white arrow). (D) Grade 2 and (E) Grade 3 enlarged lower (vagal) cranial nerve meati. (F) Assessment of Meckel’s cave long and short axis dimensions to calculate cross-sectional area. (G) Generalized effacement of the sulci (white arrows) consistent with inconspicuous subarachnoid spaces. (H, I) SSS score—a ratio of the area outlined by the white line in (H) of the superior sagittal sinus 2 cm above the torcula (white arrow) and the area outlined by the white line in (I) at the level of the cranial most aspect of the body of the corpus callosum (arrowhead). The value should be greater than 1 (wider at the torcula). (J) An arachnoid granulation (white arrow) effacing the venous signal void at the transverse/sigmoid sinus junction.

**Figure 4.**
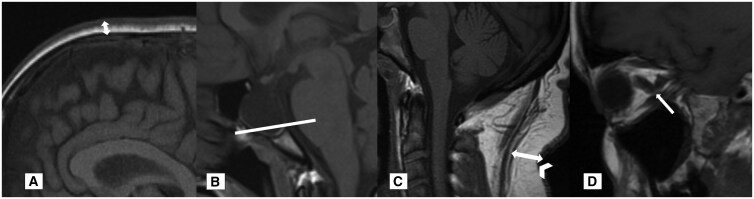
MRI measurements assessed on T1w sagittal midline imaging as illustrated in a patient with IIH. (A) Measurement of the scalp fat thickness above the coronal suture including cutaneous signal (white arrow). (B) Grade 5 Yuh and modified Yuh scores (line indicates measurement; refer to Figure 2 for grading method). (C) Measurement of C2-3 cervical fat thickness (indicated by the white arrow) and skin folding (indicated by the white arrowheads). (D) Vertical tortuosity of the optic nerve (white arrow).

Six established and 8 exploratory MRI descriptors were selected for their potential to diagnose IIH on T2w axial and T1w sagittal sequences (Table 4, Figures 2-4). Established criteria were selected from a review of the literature and had been evaluated in a cohort of IIH patients in >1 publication of diagnostic accuracy.^2^^,^^3^^,^^6-13^^,^^15^^,^^20^ These were vertical tortuosity of the optic nerves, Yuh score, inconspicuous subarachnoid spaces, enlargement of the optic nerve sheaths, globe flattening, and Meckel’s cave cross-sectional area. Exploratory descriptors were selected if they had been anecdotally linked with IIH or with similar underlying mechanisms to established descriptors or evaluating a preferential distribution of body fat which has been purported to be the case in IIH. These additional exploratory descriptors were modified Yuh score,^16^ cervical skin folding, scalp fat thickness, cervical fat thickness,^19^ superior sagittal sinus (SSS) score, widened lower cranial nerve meati, arachnoid granulations in the transverse and sigmoid sinuses, and petrous apex meningocoele.^12^

**Table 4.** MRI descriptors: description, sequence used for and method of assessment, grading (if applicable), established or exploratory feature, and the justification for selection.

MRI feature	Established or novel feature	Sequence	Method of assessment	Evaluation and grading (if applicable)	Justification for novel imaging criteria
Yuh Score	Established	Midline T1 sagittal	Classification based on the superior concavity of the pituitary gland relative to the height of the sella	See Figure 2 for grading	Patients with IIH had an 85% incidence of morphological pituitary changes.^2^^,^^6–9^
Vertical tortuosity of the optic nerve	Established	T1 sagittal (all relevant slices of the orbits)	Assessment of the optic nerve for vertical tortuosity, that is, a redundant, S-shaped appearance of the optic nerve	Yes/No for presence	Several studies have shown vertical tortuosity of the optic nerve to be associated with IIH. However, this finding has lower sensitivity than specificity.^2^^,^^6–8^^,^^10^
Inconspicuous subarachnoid spaces	Established	T2 axial	Assessment of the subarachnoid spaces for reduction in volume	Yes/No for presence	Several studies have shown low sensitivity but high specificity for IIH.^2^^,^^6^^,^^7^^,^^10^
Enlargement of the optic nerve sheaths	Established	T2 axial	Assessment of the optic nerve sheaths for enlargement of the peri-optic CSF spaces >2 mm	Yes/No for presence on either side	Several studies have shown enlarged optic nerve sheaths to be associated with IIH. However, this finding has lower sensitivity than specificity.^2^^,^^6-8^^,^^10^^,^^11^
Globe flattening	Established	T2 axial	Assessment of the posterior globe for flattening and radiological evidence of papilloedema	Grade 1: Normal Grade 2: Flattening of the posterior globe/sclera Grade 3: Optic nerve head protrusion—radiological evidence of papilloedema	Studies have shown globe flattening to be significantly associated with IIH compared with controls; however, this finding has high specificity but low sensitivity.^2^^,^^6-8^^,^^10^^,^^11^
Meckel’s cave cross sectional area	Established	T2 axial	Maximum axial long and short axis dimensions of Meckel’s cave, converted to cross-sectional area	Continuous measurement	Various studies have demonstrated Meckel’s caves to be significantly enlarged in patients with IIH.^12^^,^^13^ However, 1 study suggests narrowing of the Meckel’s cave in IIH patients.^14^ A further study suggests patients with spontaneous CSF leaks have evidence of enlarged Meckel’s cave.^15^
Modified Yuh score	Exploratory	Midline T1 sagittal	Classification based on the superior concavity of the pituitary gland relative to the height of the suprasellar cistern	See Figure 2 for grading	Including the suprasellar cistern in the sagittal cross-sectional measurement of the sella has increased sensitivity for the detection of IIH.^16^
Cervical skin folding	Exploratory	Midline T1 sagittal	Presence of ≥ 1 skin fold within the subcutaneous fat posterior to C1-C3	Yes/No for presence	IIH is associated with obesity^17^ but distribution pattern of fat may differ in IIH.^18^
Scalp fat thickness	Exploratory	Midline T1 sagittal	Thickness of the scalp fat above the coronal suture including cutaneous signal	Continuous measurement	Previously shown to be associated with IIH.^19^
Cervical fat thickness	Exploratory	Midline T1 sagittal	Thinnest dimension of posterior neck fat superior to C2-3 level between superficial fascia and including cutaneous surface	Continuous measurement	IIH is associated with obesity but the distribution pattern of fat may differ in IIH.^17^^,^^18^ One study demonstrated that cervical fat thickness is increased in IIH with an optimal threshold of 11 mm.^19^
Superior sagittal sinus (SSS) score	Exploratory	T2 axial	Measurement of the area of the SSS 2 cm above the torcula and divide by the area of the SSS at the level of the superior most aspect of the corpus callosum	Grade 1: Ratio of > 1 (SSS wider at the torcula) Grade 2: Ratio of 0.5-1 Grade 3: Ratio <0.5	Severe transverse sinus narrowing is known to be associated with IIH^2^^-4^ and similar mechanisms may be relevant to the SSS.
Widened lower cranial nerve meati	Exploratory	T2 axial	Geniculate seventh nerve, glossopharyngeal, vagal, and hypoglossal meati were reviewed. The largest was assessed.	Grade 0: Normal Grade 1: Just a slither Grade 2: Large Grade 3: Largest	Perineural CSF sleeves are associated with IIH at other cranial nerve sites (eg, optic and trigeminal nerve).
Arachnoid granulations in the transverse and sigmoid sinuses	Exploratory	T2 axial	The transverse and sigmoid sinuses were reviewed for the presence of CSF isointense arachnoid granulations indenting the flow voids in the sinuses.	Yes/No for presence and Yes/No for bilaterality	Arachnoid granulations are known to be associated with the “intraluminal” transverse sinus narrowings of IIH. Bilateral significant transverse sinus narrowings are associated with IIH.^3^
Petrous apex meningocele	Exploratory	T2 axial	Assessment of Meckel’s cave and the petrous apex for the presence of a meningocoele	Yes/No for presence	Meningocoeles are significantly more common in IIH patients.^12^

Abbreviations: CSF = cerebrospinal fluid; IIH **=** idiopathic intracranial hypertension; SSS = superior sagittal sinus.

#### Statistical analysis

Statistical analyses were performed with SPSS Statistics 29 (IBM, USA). Cohen’s kappa and intraclass correlation coefficient were performed to evaluate inter-rater reliability for the MRI descriptors.

The MRI descriptors were compared between IIH and control groups. The independent samples *t* test, Mann-Whitney-U test, and Chi-squared test were performed for normally distributed continuous MRI measurements, not normally distributed continuous MRI measurements (Shapiro-Wilk test <0.05) and categorical MRI descriptors, respectively. Statistical significance was defined as *P* < 0.004 following the Bonferroni correction.

A binomial forward stepwise logistic regression was performed to determine the optimal combination of MRI descriptors to predict IIH. According to Vittinghoff and McCulloch,^21^ the sample size allowed the inclusion of all the MRI-based predictors in the model. Multi-collinearity was evaluated with the variance inflation factor (VIF). Only variables shown to be significantly associated with an IIH diagnosis (*P* < 0.004) and VIF <5 were selected. Significant ordinal and continuous co-variates were dichotomized according to the optimal threshold values as derived from Chi squared values and receiver operating characteristic (ROC) curves, respectively. At each step, the most highly correlated variable was added, and the *P*-value threshold of 0.05 was used to set a limit on the number of variables used in the final model. Area under the ROC curve was calculated for the relevant regression model.

## Results

### Descriptive data of cohort

There were 109 IIH patients eligible according to inclusion criteria. Patients were excluded (Figure 1) leaving a cohort of 54 IIH patients (51 female, 3 male, mean age 31.2, range 15-54) and 54 controls (51 female, 3 male, mean age 31.7, range 17-41). Of the IIH patients, the mean CSF opening pressure was 38.2 ± 10.65 cmH_2_0. Presenting symptoms were headache (81.5%), tinnitus (38.9%), transient visual obscurations (25.9%), and CSF leak (otorrhea, 1.9%). Precisely, 85.2% of the cases were treated with acetazolamide. MRI was performed within 6 weeks of treatment in 30 (65%) of patients on acetazolamide.

### Inter-rater reliability

There was excellent interobserver reliability agreement with all Cohen’s kappa and intraclass correlation average measures being >0.80 except for “widened lower cranial nerve meati” with a κ = 0.75 (0.641-0.861) (Table 5).

**Table 5.** Pearson’s chi-squared test (for categorical variables) and Mann-Whitney-U test (for continuous variables including scalp fat, cervical fat thickness, and Meckel’s cave area) demonstrating correlation between MRI descriptors in the case and control groups.

MRI feature	IIH (*n* = 54)	Control (*n* = 54)	*P*-value for the statistical correlation test	Sensitivity	Specificity	Odds ratio	Interobserver reliability kappa coefficients
Vertical Tortuosity	39 (72.2%)	15 (27.8%)	**<0.004**	72.2% (58.36, 83.54)	72.2% (58.36, 83.54)	6.76 (2.91, 15.69)	0.80 (0.684, 0.908)
Inconspicuous subarachnoid spaces	4 (7.4%)	0	0.042				1
Enlargement of right optic nerve sheath	21 (38.9%)	2 (3.7%)	**<0.004**	38.9% (25.92, 53.12)	96.3% (87.25, 99.55)	16.55 (3.64, 75.24)	0.82 (0.684, 0.946)
Enlargement of left optic nerve sheath	27 (50%)	1 (1.9%)	**<0.004**	50% (36.08, 63.92)	98.1% (90.11, 99.95)	53.00 (6.83, 411.30)	0.86 (0.743, 0.966)
Globe flattening							
1	10 (18.5%)	51 (94.4%)	**<0.004**	81.5% (68.57, 90.75)	90.7% (79.70, 96.92)	43.12 (13.68, 135.92)	0.82 (0.717, 0.917)
2	25 (46.3%)	3 (5.6%)					
3	19 (35.2%)	0					
Yuh score							
1	6(11.1%)	26 (48.1%)	**<0.004**	68.5% (54.45, 80.48)	85.2% (72.88, 93.38)	12.52 (4.86, 32.21)	0.83 (0.749, 0.913)
2	11 (20.4%)	20 (37.0%)					
3	17 (31.5%)	6 (11.1%)					
4	12 (22.2%)	2 (3.7%)					
5	8 (14.8%)	0					
Meckel’s cave cross-sectional area/mm^2^	57.48 (23.21)	49.48 (14.87)	0.13				0.88 (0.830, 0.921)
Modified Yuh score							
1	5 (9.3%)	30 (55.6%)	**<0.004**	59.3% (45.03, 72.43)	88.9% (77.37, 95.81)	11.64 (4.25, 31.87)	0.80 (0.713, 0.893)
2	16 (29.6%)	18 (33.3%)					
3	14 (25.9%)	4 (7.4%)					
4	11 (20.4%)	2 (3.7%)					
5	8 (14.8%)	0					
Cervical skin folding	37 (68.5%)	10 (18.5%)	**<0.004**	68.5% (54.45, 80.48)	81.5% (68.57, 90.75)	9.58 (3.91, 23.44)	0.92 (0.851, 0.997)
Scalp fat thickness (mm)	6.11 (1.75)	5.57 (2.00)	0.14				0.95 (0.926, 0.966)
Cervical fat thickness (mm)	12.46 (3.38)	8.67 (2.75)	**<0.004**	75.9% (62.36, 86.51)	75.9% (62.36, 86.51)	9.95 (4.12, 24.04)	0.95 (0.924, 0.965)
SSS score							
1	22 (40.7)	41 (75.9%)	**<0.004**	59.3% (45.03, 72.43)	75.9% (62.36, 86.51)	4.59 (2.01, 10.49)	0.91 (0.837, 0.985)
2	28 (51.9%)	13 (24.1%)					
3	4 (7.4%)	0					
Widened lower cranial nerve meati							
0	1 (1.9%)	0	**<0.004**	28% (16.46, 41.64)	96% (87.25, 99.55)	10.00 (2.16, 46.31)	0.75 (0.641, 0.861)
1	11 (20.4%)	28 (51.9%)					
2	27 (50%)	24 (44.4%)					
3	15 (27.8%)	2 (3.7%)					
Arachnoid granulations transverse/sigmoid sinuses	10 (18.6%)	9 (16.7%)	0.68				0.84 (0.708, 0.970)
Bilateral arachnoid granulations transverse/sigmoid sinuses	3 (5.6%)	2 (3.7%)	0.65				0.82 (0.587, 1)
Petrous apex meningocoele	14 (25.9%)	1 (1.9%)	**<0.004**	25.9% (14.96, 39.65)	98.1% (90.11, 99.95)	18.55 (2.34, 146.99)	0.96 (0.882, 1)

Sensitivity, specificity, and odds ratios for optimal thresholds of significant MRI descriptors and the interobserver reliability statistics (Cohen’s kappa for categorical and intraclass correlation average measures for continuous data respectively) for the raters are stated. Data in parentheses for sensitivity, specificity, odds ratios, and interobserver reliability kappa coefficients are 95% confidence intervals. MRI descriptors with DOR > 15 are highlighted in bold type.

Abbreviations: IIH = idiopathic intracranial hypertension; SSS = superior sagittal sinus.

#### Comparison of MRI features between IIH patients and controls

IIH was associated with the 4 established MRI descriptors of vertical tortuosity, enlargement of either optic nerve sheath, globe flattening, and Yuh scores, as well as 6 exploratory MRI descriptors of modified Yuh score, cervical skin folding, cervical fat thickness, high SSS score, widened lower cranial nerve meati, and petrous apex meningocele (*P* < 0.004, Table 5).

#### Logistic regression

Nine MRI descriptors were included in the forward stepwise logistic regression analysis following exclusion of the modified Yuh score due to collinearity. Logistic regression demonstrated the 6 MRI descriptors: vertical tortuosity of the optic nerve, enlarged left nerve sheath, globe flattening score ≥ 2, Yuh score ≥ 3, cervical skin folding, and cervical fat thickness ≥ 10.5 mm to be statistically significant (χ^2^ = 102.474, *P* < 0.004). The optimal threshold for categorical variables (eg, Yuh score) was determined by establishing the highest Chi squared of different thresholds and their association with IIH. The optimal threshold for cervical fat thickness was determined by plotting an ROC curve and establishing the value with the optimal combination of sensitivity and specificity. This “optimal” model explained 82% (Nagelkerke R^2^) of the variance in IIH and correctly classified 93.5% of the cases with sensitivity 94.4%, specificity 92.6%, positive predictive value 92.7%, and negative predictive value 94.3%. The area under the ROC (AUC) was 0.965.

To provide a pragmatic model for clinical use based on a smaller number of descriptors, a post hoc logistic regression was repeated and the probability threshold (or cutoff point) was lowered from a default of 0.10 to 0.05 to reduce the number of variables that would be incorporated into the model. This “abbreviated” model demonstrated that 3 MRI descriptors were statistically significant: vertical tortuosity of the optic nerve, globe flattening score ≥ 2, and cervical fat thickness ≥ 10.5 mm (χ^2^ = 87.594, *P* < 0.001). The abbreviated model explained 74% (Nagelkerke R^2^) of the variance in IIH and correctly classified 87% of the cases with sensitivity 90.7%, specificity 83.3%, positive predictive value 84.5%, and negative predictive value 90.0%. The area under the curve was 0.947. Table 6 provides a comparison of the 2 models.

**Table 6.** Comparison of the 2 models obtained by logistic regression analysis.

	Optimal model	Abbreviated model
MRI descriptors	Vertical tortuosity of the optic nerve Enlarged left optic nerve sheath Globe flattening score ≥ 2 Yuh score ≥ 3 Cervical skin folding Cervical fat thickness ≥ 10.5 mm	Vertical tortuosity of the optic nerve Globe flattening scores ≥ 2 Cervical fat thickness ≥ 10.5 mm
*P*-value for the Omnibus tests of model coefficients	<0.004	<0.001
Nagelkerke R^2^/%	82	74
Accuracy/%	93.5	87
Sensitivity/%	94.4	90.7
Specificity/%	92.6	83.3
Positive predictive value/%	92.7	84.5
Negative predictive value/%	94.3	90.0
Area under the ROC	0.965	0.947

Abbreviation: ROC = receiver operating characteristic.

## Discussion

This study expands the range of MRI descriptors that can be usefully applied to standard MRI sequences in order to help distinguish patients with IIH. As well as corroborating established MRI descriptors, the exploratory MRI descriptors of modified Yuh score, cervical skin folding, increased cervical fat thickness, high SSS score, widened lower cranial nerve meati, and petrous apex meningocoele were demonstrated to predict IIH (*P* < 0.004) and were reliably recorded (κ = 0.75-0.95). Combining the exploratory descriptors of skin folding and cervical fat thickness ≥ 10.5 mm with the established descriptors of vertical tortuosity of the optic nerve, enlarged left optic nerve sheath, globe flattening score ≥ 2 and Yuh score ≥ 3 was able to correctly classify 93.5% of cases with AUC of 0.965. An abbreviated model combining vertical tortuosity of the optic nerve, globe flattening, and cervical fat thickness correctly classified 87% of the cases and may be a more pragmatic approach for the radiologist.

The study focused on MRI descriptors that could be analysed on standard MR T2w axial and T1w sagittal sequences. It was hoped to address the scenario whereby the radiologist needs to consider if these imaging features should prompt further investigation for IIH in a patient undergoing intracranial MRI for non-specific symptoms. Hence, some recognized MRI descriptors of IIH such as enhancement and increased DWI signal of the optic nerve head, or bilateral marked transverse sinus stenoses which cannot be applied to these sequences were not evaluated.^2^^,^^4^^,^^7^

A recent meta-analysis determined 8 MRI descriptors to have low pooled sensitivity (6.1%-68.6%) but high pooled specificity (84.0%-99.2%) for the prediction of IIH.^2^ Established imaging criteria for IIH include the association of an “empty sella” and Yuh scores with IIH.^4^^,^^6^^,^^7^^,^^9^^,^^10^^,^^16^^,^^22–24^ Yuh et al^9^ considered moderate concavity of the pituitary superior surface as the threshold of abnormality (Yuh score of 3) and established a sensitivity and specificity of 80% and 92% respectively for IIH, as compared to 68.5% and 85.2% in our study. Flattening of the posterior globe (grade 2)^2^^,^^11^ was 81.5% sensitive and 90.7% specific for IIH, as compared to a pooled sensitivity and specificity of 56.3% and 95.3% in previous studies.^2^ Vertical tortuosity of the optic nerve was 72.2% sensitive and specific for IIH vs pooled sensitivities and specificities of 36.9% and 88.4% in previous studies.^2^ Enlargement of either optic nerve sheath was 55.6% sensitive and 96.3% specific. This compares with pooled sensitivities and specificities of 68.6% and 86.1% in previous studies.^2^

With respect to the exploratory MRI features, the predictive value of the modified Yuh score^15^ achieved similar sensitivity and specificity to the conventional Yuh score (59.3% and 88.9% versus 68.5% and 85.2%) which is contrary to the previous findings of Ranganathan et al.^16^ In addition, it is of interest that cervical skin folding and mean thickness of cervical fat at C2-3 was significantly increased in IIH patients, whereas scalp fat thickness was not, suggesting a preferential distribution in body fat for the posterior cervical region in IIH patients. The optimal threshold value of 10.5 mm is similar to the 11 mm reported by Marashdeh et al^19^ in the only previous evaluation of this MRI descriptor. The intriguing finding of a narrow SSS may be a cause or result of the raised ICP in a similar way to that speculated with the better described transverse sinus stenoses.^3^^,^^4^^,^^7^^,^^22^^,^^25–28^ The advantage of this imaging sign is that it is easily recognized on routine T2w axial sequences due to the perpendicular course of the SSS to the axial plane in its distal third. The finding of widened lower cranial nerve meati may result from increased transmission of CSF in the context of chronically raised ICP in a similar way to that observed in the optic nerve sheath and Meckel’s cave.^24^^,^^29^ A single previous study found petrous apex meningocoeles in 11% of IIH patients compared to 0% in a control group^12^ as compared to 25.9% and 1.9% in our study. However, it was not possible to reproduce the findings of Degnan et al^14^ who showed the Meckel’s cave area to be narrower or Bialer et al^12^ who demonstrated them to be larger in patients with IIH.

Maintaining diagnostic specificity is particularly important due to the increasing application of MRI, with potential for overdiagnosis and excessive investigation. In this regard, it should be appreciated that IIH is a condition with low prevalence in clinical populations,^1^ and this was not reflected by our study population. The positive predictive value of the proposed MRI feature combination would be only 9.0%, assuming a prevalence of 76/100 000.^1^ Therefore, in combination with the frequently non-specific clinical presentation and uncertainty about the pre-test probability of an IIH diagnosis, there is potential to overload clinical services through unnecessary referrals. The expert clinical evaluation remains of paramount importance in triaging patients for further investigations.

Previous combinations of MRI descriptors have been tested in order to maximize sensitivity while not decreasing specificity. Examples include a study by Lim et al^30^ who reported a sensitivity of 43% and specificity of 95% for IIH when 3 or more of flattened posterior sclera, distended perioptic space, intraocular protrusion of optic nerve and empty sella were present in a paediatric cohort. Agid et al^10^ gained a marginal improvement in diagnostic accuracy relative to individual descriptors by combining empty sella with flattening of the posterior globe and achieved a sensitivity of 56.7% and specificity of 94.6%. Mallery et al^11^ showed that at least 3 of empty sella, flattening of the posterior globe, distension of the perioptic subarachnoid space, and transverse sinus stenosis provided 64% sensitivity and 100% specificity for IIH diagnosis. Finally, a study by Maralani et al^7^ determined that any combination of either partial or empty sella turcica, globe flattening, or transverse sinus stenosis was 86% sensitive and 95.3% specific. It should be noted that these latter 2 studies incorporated analysis of transverse sinus stenosis which is not possible to accurately assess on standard MRI sequences. Our data have shown that a higher diagnostic odds ratio may be achieved through the use of a wider combination of MRI descriptors available from routine MRI protocols.

A strength of this study is the relatively large sample size of 54 patients which compares favourably with 21 previous studies in a meta-analysis by Kwee et al.^2^ However, there are limitations to the study methodology which should be acknowledged. Firstly, the retrospective nature of the study resulted in a large number of excluded subjects in cases where lumbar puncture data were not available, or the MR imaging was absent, inadequate, or degraded. Ideally, results would require validation in a prospectively accrued cohort with matched control subjects. Secondly, the absence of BMI data may be considered suboptimal since it could not be applied as a co-variate or matched between the IIH and control groups. However, while some significant MRI features such as cervical skin folding and fat thickness are likely to correlate with BMI, it may be argued that the reporting radiologist rarely has access to BMI information, so it is methodologically valid to evaluate the imaging features in isolation. Thirdly, it should be noted that 44% of the IIH patients had been treated with acetazolamide prior to the MRI study which is a potential confounding factor. However, in mitigation, the characteristic MRI appearances have been shown to persist following correction of ICP.^31^ In addition, the grade of papilloedema response to acetazolamide is modest in the first 1-2 months and only 30% of patients had been treated for more than 6 weeks before the MRI.^32^ Finally, our evaluation of established MRI descriptors was not exhaustive; however, those omitted were previously noted to have <20% sensitivity for IIH (eg, cerebellar tonsillar descent and slit like ventricles).

## Conclusion

This study expands the range of descriptors that can be usefully applied to standard MRI sequences in order to distinguish patients with IIH. A combination of vertical tortuosity of the optic nerve, enlarged left optic nerve sheath, globe flattening, Yuh score, cervical skin folding, and cervical fat thickness provides optimal diagnostic performance.

## Funding

Authors acknowledge funding support from Wellcome/Engineering and Physical Sciences Research Council Centre for Medical Engineering at King’s College London (WT 203148/Z/16/Z); National Institute for Health Research Biomedical Research Centre at Guy’s & St Thomas’ Hospitals and King’s College London; Cancer Research UK National Cancer Imaging Translational Accelerator (A27066); the UK Research & Innovation London Medical Imaging and Artificial Intelligence Centre.

## Conflicts of interest

None declared.
